# Defining myocardial tissue abnormalities in end-stage renal failure with cardiac magnetic resonance imaging using native T1 mapping

**DOI:** 10.1016/j.kint.2016.06.014

**Published:** 2016-10

**Authors:** Elaine Rutherford, Mohammed A. Talle, Kenneth Mangion, Elizabeth Bell, Samuli M. Rauhalammi, Giles Roditi, Christie McComb, Aleksandra Radjenovic, Paul Welsh, Rosemary Woodward, Allan D. Struthers, Alan G. Jardine, Rajan K. Patel, Colin Berry, Patrick B. Mark

**Affiliations:** 1 Institute of Cardiovascular and Medical Sciences, BHF Glasgow Cardiovascular Research Centre, University of Glasgow, Scotland, UK; 2 University of Dundee, Division of Cardiovascular & Diabetes Medicine, Dundee, Scotland, UK

**Keywords:** cardiovascular disease, fibrosis, hemodialysis

## Abstract

Noninvasive quantification of myocardial fibrosis in end-stage renal disease is challenging. Gadolinium contrast agents previously used for cardiac magnetic resonance imaging (MRI) are contraindicated because of an association with nephrogenic systemic fibrosis. In other populations, increased myocardial native T1 times on cardiac MRI have been shown to be a surrogate marker of myocardial fibrosis. We applied this method to 33 incident hemodialysis patients and 28 age- and sex-matched healthy volunteers who underwent MRI at 3.0T. Native T1 relaxation times and feature tracking–derived global longitudinal strain as potential markers of fibrosis were compared and associated with cardiac biomarkers. Left ventricular mass indices were higher in the hemodialysis than the control group. Global, Septal and midseptal T1 times were all significantly higher in the hemodialysis group (global T1 hemodialysis 1171 ± 27 ms vs. 1154 ± 32 ms; septal T1 hemodialysis 1184 ± 29 ms vs. 1163 ± 30 ms; and midseptal T1 hemodialysis 1184 ± 34 ms vs. 1161 ± 29 ms). In the hemodialysis group, T1 times correlated with left ventricular mass indices. Septal T1 times correlated with troponin and electrocardiogram-corrected QT interval. The peak global longitudinal strain was significantly reduced in the hemodialysis group (hemodialysis -17.7±5.3% vs. -21.8±6.2%). For hemodialysis patients, the peak global longitudinal strain significantly correlated with left ventricular mass indices (*R* = 0.426), and a trend was seen for correlation with galectin-3, a biomarker of cardiac fibrosis. Thus, cardiac tissue properties of hemodialysis patients consistent with myocardial fibrosis can be determined noninvasively and associated with multiple structural and functional abnormalities.

Premature cardiovascular disease is the leading cause of death in patients with end-stage renal disease (ESRD). Excessive cardiac mortality is thought to be secondary to nonatherosclerotic processes, with sudden cardiac death being a predominant feature.^1^, ^2^ Left ventricular hypertrophy (LVH) is common in these patients and is convincingly associated with cardiovascular and all-cause mortality.^3^, ^4^, ^5^ Along with other risk factors, subclinical ischemia and hemodynamic perturbations associated with hemodialysis (HD) are likely to contribute to the ultimate development of LVH, ventricular dilation, cardiac dysfunction, and myocardial fibrosis.^6^, ^7^, ^8^ The development of these adverse features reflects a cardiomyopathy specific to uremia that develops early in chronic kidney disease (CKD).^9^, ^10^ Detection and ultimately reversal of the development of this cardiomyopathy are important goals for improving the morbidity and mortality of CKD patients.

Cardiac magnetic resonance (CMR) imaging is a useful tool in the detection of cardiac disease. One of the advantages of CMR imaging is tissue characterization.^11^ We and others previously demonstrated myocardial fibrosis in the ESRD population using contrast-enhanced CMR imaging.^9^, ^12^ More recently, imaging and quantifying myocardial fibrosis in ESRD have been challenging as gadolinium contrast agent use has been curtailed due to the association between gadolinium and nephrogenic systemic fibrosis.^13^ Noncontrast native T1 relaxation time is emerging as a viable alternative to gadolinium contrast use and has been shown to correlate with cardiac fibrosis found on tissue histology.^14^, ^15^ T1 relaxation time reflects the longitudinal recovery time of hydrogen atoms following their excitation. At any given magnetic field strength, each type of tissue will have its own normal range of values. Normal cardiac tissue will produce a specific range of values; a significant departure from the normal range is thought to represent tissue pathology.^16^ Increased native T1 time is a surrogate marker of myocardial fibrosis in other disease states such as amyloidosis and hypertrophic obstructive cardiomyopathy.^17^, ^18^, ^19^

Although increased myocardial native T1 times have been demonstrated in early CKD,^20^ until now the assessment of native T1 times in ESRD and their comparison with those in a normal healthy population has not been performed. If T1 times are greater in the ESRD population than in healthy volunteers, then they warrant further exploration to determine whether they might also be a marker of cardiac fibrosis in the renal population. Limiting the evolvement of myocardial fibrosis could be an exciting and worthwhile future end point for renal clinical trials.

In this study, we compared native myocardial global and septal T1 relaxation times as potential markers of diffuse myocardial fibrosis in incident HD patients with those of healthy volunteers (HVs). Ejection fraction is often preserved late into the development of cardiomyopathy.^21^ Myocardial strain is a more useful early marker of abnormal cardiac muscle structure and function as myocardial tissue compliance will in theory be reduced with increasing fibrosis. As echocardiographic global longitudinal strain (GLS) is predictive of histologic findings of uremic cardiomyopathy and fibrosis in rat CKD models^22^ as well as being independently predictive of increased mortality,^23^ we also compared feature-tracking CMR imaging–derived GLS between groups.

In the incident HD group, we examined the relationship between these variables and an emerging blood biomarker, galectin-3, which is potentially a surrogate of myocardial fibrosis.^24^ We also assessed the relationship between 12-lead electrocardiographic abnormalities, CMR imaging–derived cardiac indices, and other laboratory parameters including the more traditional markers of increased cardiac risk—N-terminal β-natriuretic peptide 1 (NT-proBNP) and highly sensitive troponin T (hs-tropT).

## Results

### Participants

A total of 61 aged- and sex-matched subjects were enrolled: 28 HVs and 33 HD patients. Baseline demographic characteristics of the HD population and the prevalence of traditional cardiovascular risk factors in this group are shown in Table 1. The HVs had no cardiovascular or systemic disease and had a normal electrocardiogram. They were not treated for hypertension or hypercholesterolemia and were taking no regular medications. Table 2 lists prescribed medication use by the HD patients (Table 2).

### Left ventricular mass and function

Left ventricular (LV) mass was significantly greater in the HD group: the median LV mass indexed to body surface area (LVMI) in the HD group was 69.8 g/m^2^ (interquartile range, 61.3–88) versus the HV group (55.0 g/m^2^) (interquartile range, 50.7–62.2) (*P* < 0.001). One participant in the HV group had LVH (defined as an LVMI >84.1 g/m^2^ for male participants and >76.4 g/m^2^ for female participants).^25^ In the HD group, 14 participants (42.4% of all HD participants) had LVH (*P* = 0.001).

LV ejection fraction was similar between the groups (Table 3). Five participants (15.2%) in the HD group had LV systolic dysfunction, defined as an LV ejection fraction <55%)^25^ compared with no participants in the HV group (*P* = 0.056). LV end-diastolic volumes and end-systolic volumes were similar between the groups. Full cardiac parameters of both groups are detailed in Table 3.

### Native T1 times

**Figure 1:**
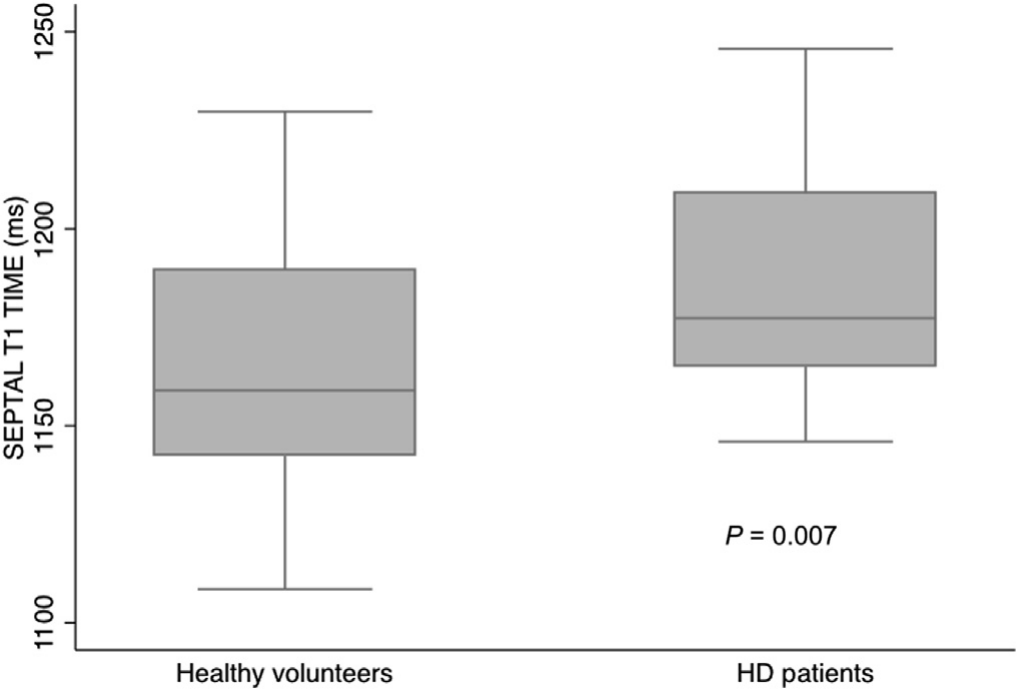
**Boxplot comparing septal T1 times in healthy volunteers and hemodialysis (HD) patients**.

Global T1 time and septal and midseptal T1 times were greater in the HD group compared with the HV group (Table 3): global T1 time in the HD group, 1171 ± 27 ms versus the HV group, 1154 ± 32 ms, *P* = 0.025; septal T1 time in the HD group, 1184 ± 29 ms versus the HV group, 1163 ± 30 ms, *P* = 0.007 (Figure 1); midseptal T1 time in the HD group, 1184 ± 34 ms versus the HV group, 1161 ± 29 ms, *P* = 0.006.

### Correlation of myocardial native T1 times (in milliseconds) with LV mass indices and function

**Figure 2:**
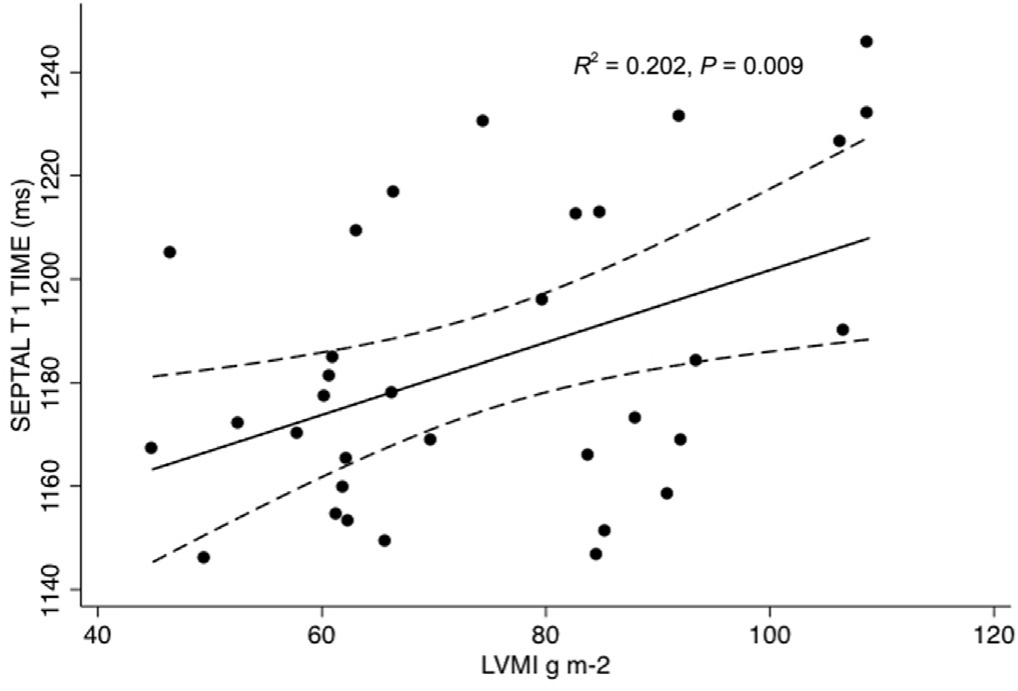
**Scatterplot of septal T1 times against left ventricular mass indexed to body surface area (LVMI) in hemodialysis patients**. g m-2, grams per meter squared.

In the HD group, LVMI correlated consistently with all measures of T1 times: Pearson’s *R* for global T1 with an LVMI = 0.452 (*P* = 0.008), for septal T1 with an LVMI = 0.449 (*P* = 0.009) (Figure 2), for midseptal T1 with an LVMI = 0.498 (*P* = 0.003). Septal T1 times positively correlated with end-diastolic volumes: Pearson’s *R* for global T1 = 0.323, *P* = 0.067, for septal T1 = 0.380 (*P* = 0.029), for midseptal T1 = 0.462 (*P* = 0.007). T1 times did not relate to ejection fraction.

### Feature tracking–derived strain

Peak GLS was reduced in the HD group compared with the HV group (HD group: GLS, −17.7 ± 5.3% vs. HV group, −21.8 ± 6.2%, *P* = 0.008).

In the HD group, GLS correlated with LVMI (Spearman’s *R* = 0.426, *P* = 0.013) and negatively correlated with ejection fraction (Pearson’s *R* = −0.535, *P* = 0.001). In the HD group, GLS also correlated with increasing end-systolic volume (Spearman’s *R* = 0.440, *P* = 0.01), which is associated with a poorer prognosis in HD.^26^

Unlike a previous study of early CKD using feature tracking–derived strain methods,^20^ we found no difference in early diastolic strain rate or strain rate between the HD and HV groups. There was no correlation between any marker of strain and native T1 values.

### Relationship of CMR imaging findings to hs-tropT, NT-proBNP, and galectin-3

In the HD group, LVMI correlated with NT-proBNP (Spearman’s *R* = 0.365, *P* = 0.044). LVMI did not correlate with hs-tropT or galectin-3. Septal T1 correlated with predialysis hs-tropT (Spearman’s *R* = 0.397, *P* = 0.027) but not with NT-proBNP or galectin-3. GLS showed a trend toward correlation with galectin-3 (Spearman’s *R* = 0.344, *P* = 0.05). There was no correlation between GLS and NT-proBNP or hs-tropT.

### 12-Lead electrocardiogram and CMR imaging findings

Twenty-eight (85%) HD participants had a pre-HD electrocardiogram available for analysis, and 27 of these subjects (96.4%) were in sinus rhythm. One participant had right bundle branch block. The median corrected QT interval was 435 ms. Significantly, there were interrelations of the Q-T interval time (QTc) with septal T1 (Spearman’s *R* = 0.376, *P* = 0.045) and NT-proBNP (Spearman’s *R* = 0.472, *P* = 0.011). There were no demonstrated relationships between LVMI or strain and QTc.

### Relationship of interdialytic fluid gains and ultrafiltration volumes with CMR imaging findings

The weight difference between the “dry weight” at the end of dialysis the day before imaging and the weight at the time of scanning correlated with LVM (but not LVMI) (Spearman’s *R* = 0.384, *P* = 0.027). There was no correlation between T1 times and this postdialysis weight gain (global T1 and prescan weight change: Spearman’s *R* = 0.052, *P* = 0.776). Strain also was not related to this weight change. An average of participants’ HD ultrafiltration volumes for the 30 days before imaging correlated with LVM, but not LVMI (Spearman’s *R* = 0.422 *P* = 0.015). There was no relationship between any measure of T1 time and 30-day mean ultrafiltration (global T1 and mean ultrafiltration Spearman’s *R* = 0.114, *P* = 0.529). There was no correlation demonstrated between any measure of strain and ultrafiltration volumes.

### Influence of blood pressure and dialysis adequacy on CMR imaging findings

In the HD group LVM (but not LVMI) correlated with diastolic blood pressure, correlation of LVM with systolic BP did not reach statistical significance (systolic blood pressure: Spearman’s *R* = 0.334, *P* = 0.057; diastolic blood pressure: *R* = 0.403, *P* = 0.02). There was no correlation demonstrated between global, septal, or midseptal T1 times or strain and blood pressure. There was a negative correlation between dialysis urea reduction ratio and LVMI (Pearson’s *R* = −0.560, *P* = 0.001). Further supporting the implication that HD adequacy was strongly correlated with LVMI, there were correlations between adjusted calcium and phosphate values and LVMI (calcium: Spearman’s *R* = −0.462, *P* = 0.007; phosphate: Pearson’s *R* = 0.358, *P* = 0.041).

T1 times did not relate to the urea reduction ratio. GLS negatively correlated with dialysis urea reduction ratios (Spearman’s *R* = −0.348, *P* = 0.047). T1 times and strain were not related to calcium or phosphate.

### Relationship of other factors known to contribute to LVH in ESRD to CMR imaging findings

HD patients with anemia (defined as hemoglobin <100 mg/dl) had a higher LVMI than those without. The median LVMI in the 6 anemic HD patients was 92.1 g/m^2^ compared with 65.7 g/m^2^ in those with a hemoglobin ≥100 mg/dl (*P* = 0.008). Our study was not powered to detect a potential small difference in T1 times in anemic patients. Median global T1 time in patients with anemia was 1190 ms (IQR, 1164.9–1209.9) versus 1171 ms (IQR, 1150.9–1187.1) (*P* = 0.189). GLS was reduced in anemic patients: GLS: anemia = −13.5 ± 3.9% versus −18.6 ± 5.1% (*P* = 0.025).

Of the HD participants, 81.8% were receiving dialysis through an arteriovenous fistula. There was no demonstrated difference in LVMI, strain, or T1 times across different types of dialysis access. No correlation was seen between age or dialysis vintage and LVMI, strain, or T1 time; 24.2% of participants were diabetic. There was no statistical difference in LVMI, any T1 measurements, or strain between those HD participants with and without diabetes.

### T1 image quality and reproducibility

**Figure 3:**
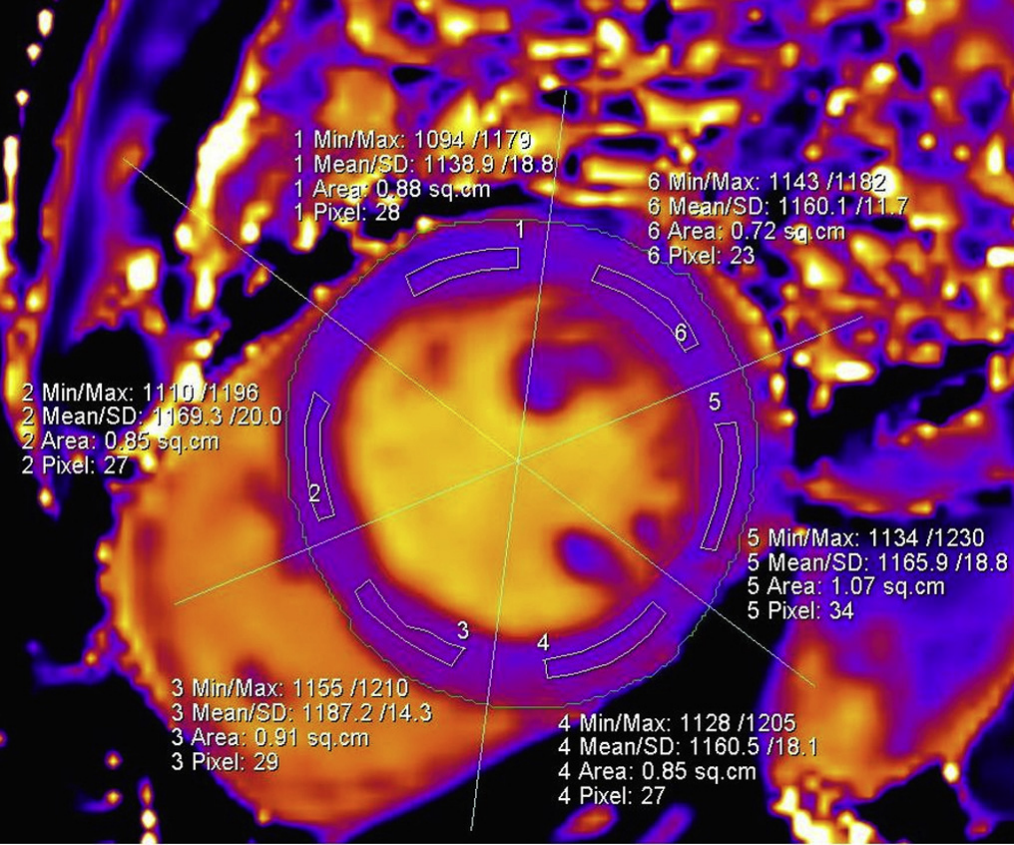
**A typically segmented T1 map of a basal myocardial slice in a hemodialysis patient.** Min/Max, minimum/maximum.

Of a total 976 T1 regions of interest (Figure 3), 808 (82.8%) were considered suitable for analysis. The greatest reliability of measurement was seen for septal T1 measurement. Septal T1 segments were less affected by artifact than other segments. The intraclass correlation coefficient for reliability of global T1 measurement was 0.872 (95% confidence interval 0.630–0.914, *P* < 0.001); for septal T1, it was 0.941 (95% CI 0.871–0.974, *P* < 0.001); and for midseptal T1, it was 0.901 (95% CI 0.786–0.955, *P* < 0.001).

## Discussion

This study is the first to compare native T1 times in HD participants with those of HVs. We demonstrated that T1 times are significantly prolonged in the HD population and correlate with increased LVMI. The increased T1 times demonstrated in the HD population may be representative of the cardiac fibrosis known to be found in ESRD. Native T1 mapping might be a novel way to quantify cardiac tissue abnormalities in HD. T1 times could be further investigated as a future surrogate end point in renal clinical trials. The utility of T1 times is further evidenced by their association with LVMI, suggesting that as LVH progresses in severity, the underlying tissue abnormalities that lead to longer T1 times increase in parallel. Although it is our assertion that these prolonged T1 times might be representative of myocardial fibrosis, without a tissue diagnosis, this cannot be proved. It should be noted that in our study that the proportion of LVH was lower than in some other HD studies. These were HD patients with a short dialysis vintage with a mean duration of renal replacement therapy of 5.5 months.

### Rationale for global and septal T1 analysis

The majority of previous studies considering native T1 times in other populations (as well as a single study in early CKD)^20^ consider increased septal T1 times to be representative of a diffuse fibrotic process. In most populations, it is possible to verify this through correlation with diffuse fibrosis seen after gadolinium contrast administration. As routine gadolinium-based contrast agent use has been curtailed in the ESRD population, verification of this assumption by this method is not practical. Additionally, although the correlation of native septal T1 times with diffuse cardiac fibrosis has also been borne out by cardiac tissue biopsy in several populations^14^, ^15^ and a previous LV biopsy study confirmed histologic evidence of diffuse fibrosis in ESRD,^27^ no study relating native septal T1 time in ESRD to cardiac tissue histology has ever been performed.

Therefore, in order to ensure that we did not falsely assume that a localized high septal T1 time was representative of a diffuse process, we measured T1 times throughout 3 short-axis slices: basal, mid, and apical. Using the 16-segment model of the American Heart Association,^28^ we calculated an average global T1 time from all 16 regions of interest. Using this method, we were able to demonstrate that truly global T1 times were higher in the HD population than in healthy controls. Global T1 times were very closely correlated with septal T1 times (Pearson’s *R* = 0.885, *P* < 0.001). We analyzed midseptal T1 times alone on the assumption that the apical short-axis slice might be more susceptible to motion artifact than the midslice; however, midseptal T1 times were not of any greater value than septal T1 time. Furthermore, using only 2 segments to calculate midseptal T1 increases the risk of regional ischemia influencing results.

### Influence of traditional ischemic heart disease on T1 times

Native T1 times are known to be locally higher in areas of cardiac injury, for example, after a chronic myocardial infarction.^29^ Our HD population was unselected, and a proportion of patients had known ischemic heart disease. Before any knowledge of the association of gadolinium contrast use and nephrogenic systemic fibrosis in ESRD, it was described using gadolinium contrast that there are 2 patterns of cardiac fibrosis seen in ESRD: that of the traditional ischemic heart disease with subendocardial fibrosis and that of a more diffuse uremic cardiomyopathy.^9^, ^12^ We do not believe that the prolonged T1 times in the HD group were a consequence of traditional ischemic heart disease. Four HD participants had a history of myocardial infarction. Two observers independently reviewed cine images of these participants to exclude any patients who had thinned akinetic myocardial segments, which is commonly accepted as a surrogate of transmural chronic myocardial infarction. The observers agreed that there was no evidence of myocardial wall thinning on any of their images. Furthermore, if these 4 participants were excluded entirely, then the average global, septal, and midseptal T1 times for the HD group were essentially unaltered (global T1 with myocardial infarction patients excluded = 1170 ± 27 ms vs. 1171 ± 27 ms with myocardial infarction patients included).

### Arrhythmia, hs-tropT, and T1 time

Interestingly, we saw a relationship between septal T1 and predialysis hs-tropT. Septal times also related to QTc. Increased corrected Q-T interval times are associated with an increased risk of sudden cardiac death.^30^ The increased incidence of sudden cardiac death is a poorly understood phenomenon in ESRD that cannot be entirely attributed to electrolyte disturbances.^30^ A study in rats showed that rats with induced CKD develop both LVH and increased susceptibility to arrhythmia.^31^ In this study, there were also some early signs that cardiac fibrosis began to develop in the rats. The demonstrated correlation between septal T1 times and troponin T and septal times and QTc in our study warrants further exploration.

### GLS, ejection fraction, and early myocardial dysfunction

Our study showed no difference in ejection fraction between HV and HD groups. It is well-known that ejection fraction is often preserved until late in the development of cardiomyopathy, and thus it is not a reliable primary end point for renal clinical trials.^21^ Recent studies showed that echocardiographic myocardial strain is an independent predictor of increased mortality in CKD populations.^22^, ^23^

In this study, there was a trend toward a correlation of GLS with galectin-3 (Spearman’s *R* = 0.344, *P* = 0.05). Given our small sample size, we consider this result to be of some interest. Galectin-3 is an established biomarker of myocardial fibrosis. The trend identified makes sense as GLS measured by speckle tracking echocardiography is predictive of histologic findings of uremic cardiomyopathy and cardiac fibrosis in rats with CKD.^22^

Assessment of GLS using speckle tracking echocardiography is recommended in recent guidelines for the quantitative assessment of LV function.^32^ However, echocardiography is not an ideal tool for clinical trials in ESRD because dialysis-associated fluid shifts can lead to overestimation of ventricular indices and variations in assessment of cardiac function.^33^ Use of a single imaging modality to assess outcomes in the HD population is preferable, and we consider CMR imaging to be the modality of choice. In particular because, more recently, myocardial strain quantified by CMR imaging has been found to be associated with adverse outcomes.^34^ Although there was no correlation of T1 times with GLS, our findings of a difference in GLS between the HD and HV groups, as well as the correlation of GLS with LVM in HD, support the assertion that altered myocardial strain mechanics have some role in the process of development of LVH and potentially fibrosis in CKD patients. Whether abnormal GLS is a cause or a consequence of cardiomyopathy development needs to be further investigated. Similarly, the lack of a correlation of T1 times with GLS in this study should be considered further. At present, it is not clear whether our study was underpowered to detect a relationship between the 2 or whether they reflect slightly different aspects of pathologic processes in the heart. In any case, they both have their own merits; GLS is a dynamic measure, whereas T1 time is arguably a more fixed quantity.

### Limitations

Although our study numbers are relatively small and all participants came from a single center, we have been able to present a well-characterized group of dialysis patients with a short dialysis vintage. The incidence of diabetes in our study population (24.2%) was less than that in many typical HD populations, and our study population was not racially diverse. We were unable to perform multivariable adjustments to assess for independence of any of our findings.

Our study would have been strengthened by the addition of a control population of hypertensive patients with LVH and without renal disease. However, there is some evidence from other studies that included a control group that in contrast to LVMI, T1 times are independent of blood pressure.^20^ The fact that T1 times and strain were not related to blood pressure in our study provides some further reassurance in this regard.

Without biopsy confirmation, we cannot be certain that the cardiac abnormalities identified in this population are representative of fibrosis. It is recognized that water content can prolong T1 times, and this is undoubtedly a limitation of our study as we cannot be sure of the precise influence of this on our results.^35^ However, we saw no correlation with weight gain between HD and imaging with T1 times. Similarly, there was no correlation between T1 time and ultrafiltration volumes. Both of these markers, which are representative of changing fluid status, correlated with LVM. Overall, we believe that these findings support our assertion that increased T1 times in the HD population are likely to reflect tissue abnormalities (potentially fibrosis). Previous biopsies of the uremic hearts have shown extensive myocardial fibrosis, which would be consistent with our conclusions.^27^

### Implications of our study findings

T1 times are a potentially novel way to quantify cardiac tissue abnormalities in ESRD. In the future, they may be conclusively demonstrated to be a surrogate marker of fibrosis. Nephrologists should note that normal values will vary from scanner to scanner. The next steps for development of T1 mapping as a widely applicable clinical tool will involve each center developing robust normal values for each of their scanners. Phantom work will be required before any future multicenter collaborations run successfully.

We showed that T1 times are abnormally high in the HD population. Perhaps in the future, change in T1 times could potentially be a primary outcome measure in renal clinical trials. However, a number of questions need to be answered before this can happen: what is the natural history of T1 times throughout the progression of CKD, do T1 times change after renal transplantation, how exactly are T1 times affected by fluid status variation, and are increased T1 times associated with increased risk of future cardiovascular events and mortality. To date, increased T1 times have been shown to be predictive of mortality in amyloidosis^36^; the utility of T1 times to predict mortality in other populations remains to be proved.

## Methods

### Participants

Sixty-one participants were included in the study. All participants were older than 18 years of age and had no contraindications to CMR scanning. HD participants were eligible if they had commenced dialysis within the past 12 months; baseline scans for all eligible patients in 2 concurrent studies recruiting in Glasgow were used. In total, 33 HD patients were recruited from the Cardiac Uraemic fibrosis Detection in DiaLysis patiEnts study (CUDDLE study, ISRCTN99591655), and the ALTERED study (Does ALlopurinol Regress LefT Ventricular Hypertrophy in End Stage REnal Disease, NCT01951404). A total of 28 age- and sex-matched healthy volunteers were recruited from a Glasgow-based study examining variations in native T1 relaxation times in healthy adults.^37^ Healthy volunteers within 5 years of age of each HD participant were blindly selected from a list of HV participants that detailed only the HV participants’ age and sex.

All participants provided written informed consent, and all studies were approved by local ethics committees (CUDDLE: West of Scotland Research Ethics Service, reference 13/WS/0301, healthy volunteers: 11/AL/0190; ALTERED: East of Scotland Research Ethics Service, 13/ES/0051).

### Inclusion and exclusion criteria

The HV group had no cardiovascular or systemic disease and had normal electrocardiograms. They were not treated for hypertension or hypercholesterolemia and were taking no regular medications. HD patients were excluded if they had atrial fibrillation (as this makes magnetic resonance imaging and electrocardiographic gating difficult). All HD patients had been receiving renal replacement therapy for less than 1 year. ALTERED patients were not taking allopurinol, had a life expectancy >1 year, and did not have echocardiogram-defined LV systolic dysfunction.

### Magnetic resonance image acquisition

All participants underwent 3-T CMR imaging (MAGNETOM Verio, Siemens Healthcare, Erlangen, Germany) at the British Heart Foundation Clinical Research Centre, University of Glasgow. In the HD patients, CMR imaging was consistently performed on a postdialysis day. Baseline ALTERED study magnetic resonance imaging scans were used before any study intervention. A double radiofrequency coil array (anterior and posterior) was used. The scans were electrocardiographically gated. The imaging protocol included cine magnetic resonance with steady-state free precession and T1 mapping sequences.^35^ The full left ventricle was captured in a cine short-axis stack. Cine acquisition parameters included 41.4-ms repetition time, 40° flip angle, 1.51-ms echo time, 256 × 173-pixel matrix, 1.5 × 1.3 × 8-mm voxel size, 8-mm slice thickness, and 977-Hz/pixel bandwidth.

Basal, mid, and apical T1 maps were acquired in 3 short-axis slices using a motion-corrected, optimized, modified Look-Locker inversion recovery investigational prototype sequence without contrast administration (Siemens Healthcare, works-in-progress method 448). T1 imaging parameters included 267.84-ms repetition time, 35° flip angle, 1.06-ms echo time, 100-ms T1 of the first experiment, 80-ms T1 increment, 124 × 192 pixel matrix, 2.2 × 1.8 × 8.0-mm spatial resolution, 930-Hz/pixel bandwidth, and 17-heartbeat (range, 12–18 s) scan time.

### Image analysis

#### LVM and function

A single observer analyzed anonymized images in a random order to determine cardiac indices including LVM and function by manually tracing endocardial and epicardial borders at end-systole and end-diastole on the short-axis cine images as according to well-established protocols.^37^ End-systolic and end-diastolic volumes and LVM were calculated and indexed to body surface area (using a weight acquired immediately prescan) using the Siemens Argus analysis software.

#### T1 maps

On the raw T1 images, LV contours were defined and copied onto the color-enhanced spatially coregistered maps. Using the anterior right ventricular-LV insertion post as a reference, T1 maps were segmented according to the American Heart Association 16-segment model.^28^ Segmental American Heart Association regions of interest were delineated by user-defined semiautomated border delineation (Siemens Argus analysis software). The regions of interest were standardized to be of similar size and shape. T1 times were measured in each of the 16 segments, with care taken to delineate regions of interest with adequate margins of separation from tissue interfaces such as between the blood pool and myocardium. A typical T1 map is shown in Figure 3.

Each individual segment was assessed for the presence or absence of susceptibility and motion artifacts. After removal of any segments affected by artifact, a global T1 time was calculated from the mean of all remaining segments. A septal T1 time was calculated by averaging the acceptable anteroseptal, inferoseptal, and septal American Heart Association segments (segment numbers 2, 3, 8, 9, and 14). Midseptal T1 time was derived from an average of included septal segments from the midshort-axis slice (segment numbers 8 and 9). T1 map reproducibility was assessed by blinded reanalysis of 25 randomly selected images by the same observer.

#### Strain

Dedicated feature-tracking software (Diogenes Image Arena, Munich, Germany) was used on the horizontal long-axis cine acquisition. In a method previously described,^38^, ^39^ the end-diastolic frame was identified for each image, and endocardial borders were delineated. The delineated contour was then automatically propagated throughout the cardiac cycle. GLS, strain rate, and early diastolic strain rate were then calculated. Intraobserver reproducibility was checked by blinded reanalysis of a proportion of images (*n* = 20).

### 12-Lead electrocardiograms

In the HD group, predialysis 12-lead electrocardiograms were obtained for 28 participants. The underlying rhythm was recorded. Corrected QTc was noted from the electronic print out of each electrocardiogram.

### HD patient biomarkers and other clinical parameters

Frozen stored predialysis blood samples were obtained in the HD group for analysis of hs-tropT, NT-proBNP (e411, Roche Diagnostics, Burgess Hill, UK), and galectin-3 (Bio-Techne, Abingdon, UK). All were analyzed using the manufacturer-recommended protocols and calibrations. Available collected blood tests from the time of imaging, including hemoglobin, urea reduction ratios, albumin, C-reactive protein, phosphate, parathyroid hormone, glucose, predialysis creatinine and potassium, lipid profile, as well as each HD participant’s medical and dialysis history including ultrafiltration volumes and postdialysis weights were obtained from electronic records.

### Statistics

All statistical analyses were performed using SPSS version 22 (Armonk, NY) and STATA 13 (StataCorp, College Station, TX). Paired *t* tests (for parametric data) and Mann-Whitney *U* tests (for nonparametric data) were used to compare continuous indices between HVs and HD patients. Categorical data were assessed using the χ^2^ test or the Fisher exact test, as appropriate. Correlations within each group between continuous indices were assessed using Pearson’s and Spearman’s correlation coefficients for parametric and nonparametric data, respectively.

At the time of the start of this study, there were few data to inform a power calculation using native T1 times at 3-T CMR imaging. Based on our normal volunteer study,^37^ to detect a 30-ms difference in mean native T1 time between a group of HVs and patients with ESRD, at 90% power and a probability of a type 1 error of 0.05 would require 22 per group, based on a standard deviation in T1 time of 30 ms in each group.

## Disclosure

The University of Glasgow holds a research agreement with Siemens Healthcare (UK). All the other authors declared no competing interests.

## Figures and Tables

**Table 1:** Baseline Demographic Characteristics and Clinical Data for HD Patients

Variable	All HD Patients (N = 33)
Primary renal diagnosis (%)	
Diabetic nephropathy	24.2 [8]
Polycystic kidney disease	15.2 [5]
Renovascular disease	12.1 [4]
Glomerulonephritis	24.2 [8]
Unknown cause	12.1 [4]
Other known	12.1 [4]
Length of time on HD (mo)	5.5 ± 2.7
Mean UF volume (l)	1.7 ± 1.0
Dialysis access (%)	
Fistula	81.8 [27]
Graft	3 [1]
Tunneled line	15.2 [5]
Diabetes [%]	24.2 [8]
Hypertension [%]	60.6 [20]
Myocardial infarction [%]	12.1 [4]
Ischemic heart disease [%]	18.2 [6]
Stroke [%]	15.2 [5]
Peripheral vascular disease [%]	9.1 [3]
Systolic blood pressure (mm Hg)	138 (131–155)
Diastolic blood pressure (mm Hg)	78 (67–83)
Hemoglobin (mg/dl)	111 (103–118)
Urea reduction ratio (%)	73 (68–78)
Albumin (g/l)	35 (32–36)
C-reactive protein (mmol/l)	8 (3–14)
Phosphate (mmol/l)	1.73 (1.33–2.18)
Corrected calcium (mmol/l)	2.35 (2.24–2.39)
Parathyroid hormone (mmol/l)	48.1 (24.5–85.9)
Galectin-3 (ng/ml)	17.5 (13.4–22.0)
hs-Troponin T^a^ (pg/ml)	33.7 (23.5–46.9)
NT-BNP^a^ (pg/ml)	1934 (1111–5111.5)
ECG QTc^b^ (m/s)	435 (412.8–453)

ECG, electrocardiogram; HD, hemodialysis; hs-Troponin T, highly sensitive Troponin T; NT-proBNP, N-terminal β-natriuretic peptide 1; UF, ultrafiltration.

All data presented as percentage [number of participants], median (interquartile range), or mean ± SD, as appropriate.

a hs-Troponin T and NT-BNP values were available for 31 HD participants.

b QTc was available for 28 HD participants.

**Table 2:** Prescribed Medications in the HD Participants

Medication	HD Participants Taking (*N* = 33)
Erythropoietin	78.8 (26)
Beta-blocker	57.6 (19)
Aspirin	24.2 (8)
Clopidogrel	24.2 (8)
ACE inhibitor	9.1 (3)
Diuretics	27.3 (9)
Calcium channel blockers	39.4 (13)
Alpha-blockers	6.1 (2)
Statin	54.5 (18)

ACE, angiotensin-converting enzyme; HD, hemodialysis.

Data shown as percentage (number of participants).

**Table 3:** Patient Characteristics and Cardiac Parameters of HV and HD Patients

Variable	Healthy Volunteers (*N* = 28)	HD Patients (*N* = 33)	*P* Value
Age (yr)	60 (53.8–72.3)	56 (50–71)	0.562
Male [%]	57.1 [16]	57.6 [19]	0.973
Weight (kg)	79 (68.8–89)	73.9 (63–83)	0.343
BMI (kg/m^2^)	25.6 (24.1–29.5)	27.7 (23.1–30.5)	0.772
Ethnicity			
White	96.4 [27]	90.9 [30]	0.618
South Asian	3.6 [1]	9.1 [3]	0.618
Global T1 (ms)	1154 ± 32	1171± 27	0.025
Septal T1 (ms)	1163 ± 30	1184 ± 29	0.007
Midseptal T1 (ms)	1161 ± 29	1184 ± 34	0.006
LVM (g)	107.7 (89.9–115.6)	131.7 (105.8–152.6)	0.001
LVMI (g/m^2^)	55.0 (50.7–62.2)	69.8 (61.3–88)	0.001
LVH (%)	3.6 [1]	42.4 [14]	0.001
EDV (ml)	148.4 (128.2–168.9)	142.9 (133.6–163.8)	0.856
EDVI (ml/m^2^)	77.4 ± 9.7	83.8 ± 23.4	0.180
ESV (ml)	56.8 (43.4–62.4)	51.2 (41.8–64.1)	0.783
ESVI (ml/m^2^)	28.4 ± 6.0	32.0 ± 17.5	0.307
LV Dilation	0 [0]	9.1 [3]	0.243
Stroke Volume (ml)	94.4 (75.8–103.4)	92.8 (74.2–112.2)	0.954
Ejection Fraction (%)	63.3 ± 5.2	63.2 ± 9.3	0.963
LVSD (%)	0 [0]	15.2 [5]	0.056
GLS (%)	-21.8 ± 6.2	-17.7 ± 5.3	0.007
Strain Rate (s^−1^)	1.06 ± 0.37	0.95 ± 0.24	0.140
EDSR (s^-1^)	0.97 ± 0.36	1.03 ± 0.36	0.473

BMI, body mass index; EDSR, early diastolic strain rate; EDV, end-diastolic volume; EDVI, end-diastolic volume indexed to body surface area; ESV, end-systolic volume; ESVI, end-systolic volume indexed to body surface area; GLS, global longitudinal strain; HD, hemodialysis; HV, health volunteer; LV dilation, left ventricular dilation; LVH, left ventricular hypertrophy; LVM, left ventricular mass; LVMI, left ventricular mass indexed to body surface area; LVSD, left ventricular systolic dysfunction.

All data are shown as mean ± SD, median (interquartile range), or percentage [number of participants], as appropriate.
